# Hyperferritinemic Sepsis: An Opportunity for Earlier Diagnosis and Intervention?

**DOI:** 10.3389/fped.2016.00077

**Published:** 2016-08-02

**Authors:** E. Scott Halstead, Surender Rajasekaran, Julie C. Fitzgerald, Scott L. Weiss

**Affiliations:** ^1^Division of Pediatric Critical Care Medicine, Department of Pediatrics, Pennsylvania State University College of Medicine, Hershey, PA, USA; ^2^Pediatric Critical Care Medicine, Helen DeVos Children’s Hospital, Grand Rapids, MI, USA; ^3^Department of Anesthesiology and Critical Care Medicine, University of Pennsylvania Perelman School of Medicine, Philadelphia, PA, USA

**Keywords:** sepsis, hemophagocytic lymphohistiocytosis, macrophage activation syndrome, HSV, ferritin, cytokine storm

## Abstract

We describe a case of an infant with HSV meningitis and septic shock who demonstrated a remarkably high serum ferritin level. Aggressive pediatric intensive care and the administration of high-dose glucocorticoids were not able to reverse the multiple organ dysfunctions. Subsequent autopsy identified the presence of hemophagocytosis, thus the patient fulfilled hemophagocytic lymphohistiocytosis (HLH) criteria post-mortem. This case highlights that serum ferritin may be an important early indicator of mortality in sepsis due to a cytokine storm similar to macrophage activation syndrome and HLH.

## Case Report

Patient A, a 5-day-old infant female, 2.2 kg, born prematurely at 35 weeks gestational age, was transported to an outside hospital emergency room *via* emergency medical services following an apneic event at home that resolved after the administration of a few rescue breaths by the mother. Upon presentation to the emergency room, the patient demonstrated stable vital signs and was subsequently transferred to our pediatric intensive care unit (PICU) for respiratory monitoring and diagnostic workup for the cause of the apneic event. Upon arrival to the PICU, the patient was vigorous, crying, and tachypneic, but, otherwise, with relatively normal vitals signs (HR 145, BP 77/44, 97% on 1 LPM NC O2). Given the patient’s age and history of apnea, a workup to rule out serious bacterial infection was initiated. Basic labs were sent, and a lumbar puncture was completed showing 20 WBC/μL (2,030 RBC/μL) with 33% neutrophils. Broad-spectrum antimicrobials were initiated including ceftriaxone (50 mg/kg/dose prior to arrival to PICU), acyclovir (60 mg/kg/day), and ampicillin (400 mg/kg/day). Initial labs were significant for thrombocytopenia (platelets 39,000/μL) but without any other cytopenias (Hgb 17.1 g/dL, WBC 8,760/μL).

At hour 6 after admission, the patient rapidly decompensated and developed lethargy, respiratory failure due to apnea, and was intubated and mechanically ventilated. At hour 10 after admission, additional laboratory testing revealed very elevated liver transaminases (ALT 2,214, AST > 7,500), severe coagulopathy (PT > 120, INR > 16.6, PTT > 200) and a ferritin of 241,000 ng/mL (result verified with duplication). The patient’s triglyceride level was 41 mg/dL, and serum lactate level at this time was significantly elevated at 14 mmol/L.

**Figure 1:**
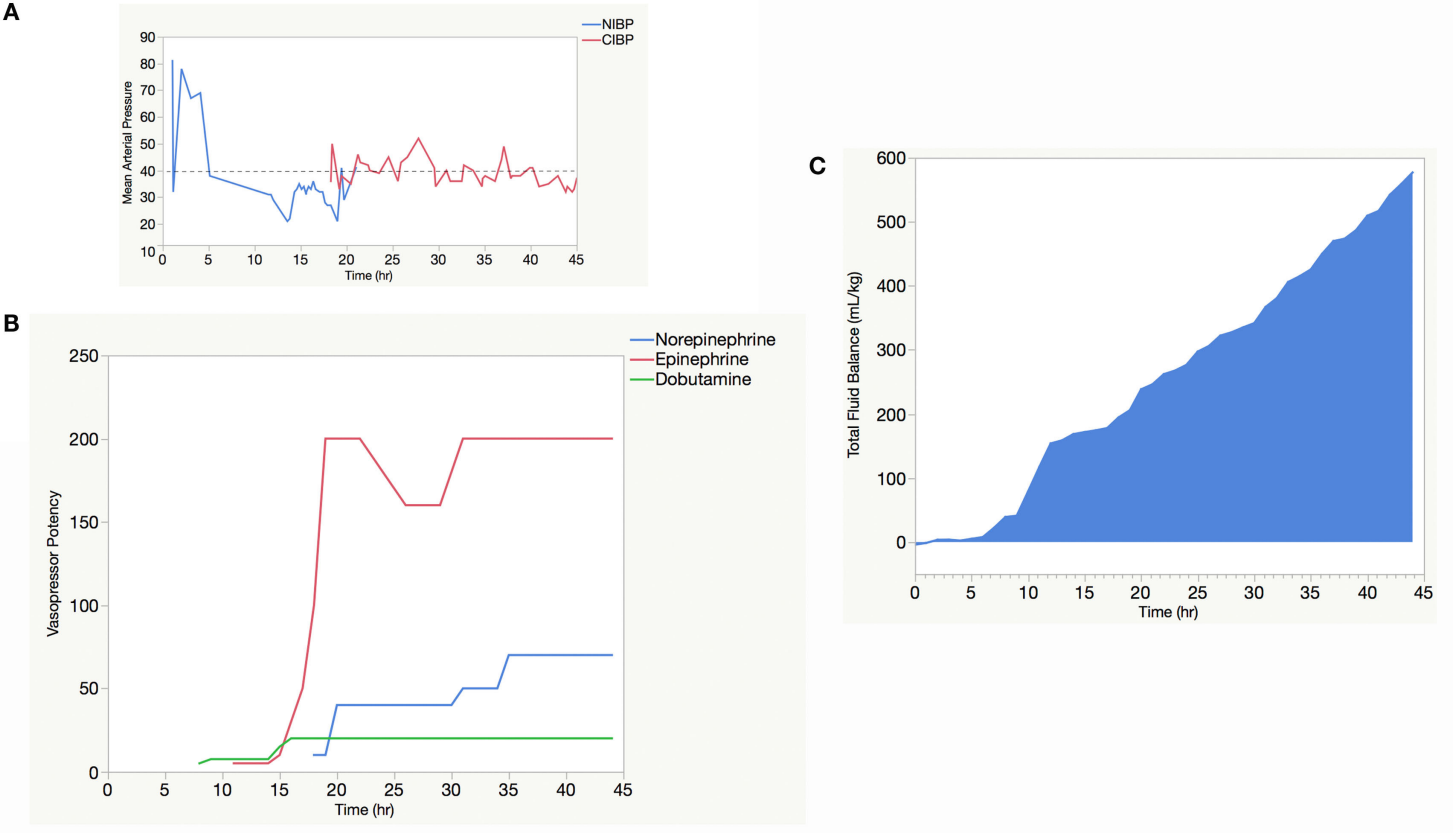
**Vasopressor and fluid resuscitation support needed to maintain a minimum mean arterial pressure (MAP) of 40 mmHg**. The kinetics of the mean arterial blood pressure as determined by non-invasive blood pressure (NIBP) “cuff” (blue line) and continuous invasive blood pressure (CIBP) “arterial line” (red line) **(A)**. The dosing of vasopressors, expressed as vasopressor potency, against time **(B)**. Given their higher biologic activity, vasopressor potency for epinephrine (red line) and norepinephrine (green line) were calculated as the dose (μg/kg/min) × 100, whereas the vasopressor potency of dobutamine (blue line) was calculated as simply the dose of dobutamine (microgram per kilogram per minute). Ongoing fluid resuscitation using both colloid (5% albumin in NS) and blood products (including packed red blood cells and fresh frozen plasma) needed to maintain a minimum MAP of 40 mmHg (dotted line) **(C)**.

At hour 18 after admission, the HSV PCR from CSF returned positive for HSV-2, and the C-reactive protein (CRP) was elevated (2.09 mg/dL). We made the preliminary diagnosis of HSV-associated hemophagocytic lymphohistiocytosis (HLH) based on a similar case report (1). The patient’s respiratory and hemodynamic profile continued to deteriorate (Figure 1A) in spite of escalating vasopressor support (Figure 1B), and the patient needed repeated fluid boluses of colloid (5% human serum albumin in NS) and blood product administration (Figure 1C) to maintain mean arterial pressure (MAP) of at least 40 mmHg. At this time, 30 mg/kg methylprednisolone was administered to ameliorate the inflammatory process and, with the addition of norepinephrine, the patient stabilized transiently, and we were able to wean the epinephrine support slightly (Figure 1B).

At hour 30 after admission, the patient again became unstable and required repeated volume resuscitation in the form of colloid fluid boluses to maintain the MAP goal of 40 mmHg, and then, even this MAP goal became unobtainable. After long discussion with the parents, it was decided that recovery was unlikely and technological support was withdrawn. The parents agreed to an autopsy, and the report stated that “the presence of hemophagocytosis was demonstrated with the bone marrow and spleen exhibiting large cells containing particles consistent with ingested, disintegrating blood cells. Given that diagnostic guidelines for HLH designate bone marrow, lymph node, and spleen as the organs, which most reliably show the histologic features, and secondary HLH can be triggered by systemic infection, as this patient had, the findings post-mortem supported a diagnosis of HLH.”

## Discussion

We find this case to be of interest given the rapid deterioration of a patient with HSV-associated multiple organ dysfunction syndrome (MODS). Unfortunately, delivery of anti-inflammatory treatment did not significantly affect the outcome. Historically, the patient would have been diagnosed with neonatal HSV-associated fulminant hepatitis (2, 3). However, the patient also had a very elevated ferritin level of 241,000 ng/mL. This led us to preliminarily diagnose the patient with HSV-associated HLH (4). Of the eight criteria proposed in the HLH-2004 guidelines (5), our patient met four out of the five requisite criteria for a diagnosis of HLH:

1. Temperature instability (Patient’s T_min_ 35.9°C)
2. Hypofibrinogenemia (Patient’s fibrinogen = 94 mg/dL)
3. Hyperferritinemia (Patient’s ferritin = 241,000 ng/mL)
4. Cytopenias (affecting ≥2 out of 3 lineages in the peripheral blood)
5. Platelets <100 × 10^9^/L (Patient’s Plts = 38 × 10^9^/L)
6. Hemoglobin <9 g/dL in infants <4 weeks of age (Patient’s Hgb = 8.1 g/dL).

It is notable that the patient did not have anemia when she was first admitted. However, her hemoglobin fell precipitously and was maintained at approximately 10 g/dL with packed red blood cell (pRBC) transfusions to maintain oxygen carrying capacity in light of an ongoing lactic acidosis. The soluble interleukin (IL)-2 receptor (sCD25) was sent 28 h after admission and showed a value of 755 pg/mL (85.3 U/mL), far less than the 2,400 U/mL cut-point defined by HLH-2004 diagnostic criteria. Natural killer (NK)-cell activity was not performed though it would be expected to be low (6). Furthermore, hemophagocytosis was seen on autopsy. Therefore, if one includes the hemophagocytosis seen on autopsy, this patient met HLH criteria, and the high dose pulse of methylprednisolone seems to have been appropriate. Interestingly, following the high dose pulse of methylprednisolone (30 mg/kg), the ferritin fell to 33,600 ng/mL and the CRP to 0.89 mg/dL at 38-h post-admission.

In terms of risk factors for familial HLH, the parents were neither related nor were family histories of early unexpected death obtained. While not frequently described, case reports reveal that hyperferritinemia may be present in disseminated neonatal HSV infection. Yamada et al. described a 4-day-old male infant with HSV-I infection and a ferritin of >15,000 ng/mL, who seemed to respond to methylprednisolone (30 mg/kg/day), intravenous immunoglobulin (IVIG, 1 g/kg/day), and whole blood exchange transfusion, who recovered completely (1). Imashuku et al. described three neonatal patients (age 3–14 days), who they described as fulfilling diagnostic criteria for HLH, who also had HSV infection, and who all died as a result of MODS (4). Vladescu et al. recently described three neonatal patients (age 6–10 days) with HSV infection and hyperferritinemia (>40,000–68,090) (7). Of these three patients, the only patient to survive was the patient who fulfilled 6 out of 8 criteria for HLH but was not started on HLH-2004 therapy due to her improving condition on acyclovir alone (7).

These cases invite speculation that hyperferritinemia may be a feature of disseminated HSV infection. So, what is the reason for the hyperferritinemia during HSV infection and for that matter, HLH/macrophage activation syndrome (MAS)? Ferritin is a key molecule that serves to limit pro-oxidant stress that typifies inflammatory conditions (8), and the major source of serum ferritin is tissue macrophages (9). Interestingly, pediatric patients with ferritin levels greater than 3,000 ng/mL have an increased risk of death (10). While the details are unclear, ferritin expression by macrophages is induced by pro-inflammatory cytokines including IL-1, IL-6, IL-12, IL-18, interferon-gamma (IFN-γ), and tumor necrosis factor alpha (TNF-α) (11). Of course, from a pediatric intensivist’s perspective, while the cause of the cytokine storm is important, stopping it is essential.

Various strategies and therapeutic regimens have been applied to HLH and MAS. In 1994, the Histiocyte Society published a protocol with the recommendation of an etoposide and dexamethasone-based regimen, which remains the basis of initial therapy for HLH, with definitive therapy being hematopoietic cell transplantation (HCT) (12). For genetic deficiency-verified familial HLH, this approach seems very reasonable. However, at the present, genetic verification takes weeks, and the administration of cytotoxic agents to patients with fulminant shock is potentially risky. In our case, the hematology/oncology team was comfortable with high dose steroids but wanted further lab results prior to committing to the etoposide-based regimen. However, etoposide may have a role in the acute setting of shock and MAS/HLH as a preclinical mouse model has shown that it selectively depletes activated T lymphocytes (13). To the authors’ knowledge, the safety of etoposide administration in a patient with fulminant shock has not been studied. Another immuno-modulatory approach, therapeutic plasma exchange (TPE) coupled with IVIG and methylprednisolone, has been used clinically (14). Given the patients’ small size (2.2 kg), placement of an apheresis catheter was not attempted and, therefore, TPE was not performed. IVIG administration was considered and ordered, but the decision to withdrawal of technological support was made before it could be administered.

The hyperferritinemia of MAS/HLH most likely represents a hypercytokinemic storm, and the targeting of the component cytokines, including IL-1, IL-6, and IFNγ, is becoming a target of active research (15). In fact, anakina (Kineret), an IL-1 receptor antagonist, has been used successfully in similar cases of secondary HLH/MAS/MODS (16). In our case, anakinra was not on formulary and was unavailable. Ultimately, increased specificity and expedited delivery of the diagnosis will be vital to answer the question as to the most appropriate therapy. Indeed, much of the historical discussion in the literature up to this point regarding HLH, MAS, and hyperferritinemic sepsis-related MODS has revolved around making the distinction of familial versus secondary HLH, and the difference in treatment between the two. However, may be it is time to change the paradigm of the discussion and, instead, generate a more inclusive criterion that would speed diagnosis and treatment of the cytokine storm first, with later determination of the cause of the storm. Such a broadened criterion may be hyperferritinemic sepsis (HFS), with or without MODS. The broadened HFS criterion may expedite diagnosis as it would decrease the initial number of hard to obtain laboratory values, and, instead, the preliminary criteria would be based on clinical findings and an elevated ferritin value alone. It would also help clinical research, as the criteria for HLH are strict. The simplified criteria for HFS may increase the number of patients who would meet eligibility criteria for initial anti-inflammatory therapy. While increasing sensitivity, the use of HFS criterion would decrease diagnostic specificity of HLH. However, a tiered approach to treatment could be undertaken, allowing for earlier therapy initiation while diagnostic testing is pending. Absence or presence of MODS would impact the risk benefit ratio and treatment decisions regarding more aggressive therapies including cytokine blockade (16) or TPE (17), and subsequent results of genetic testing would dictate the later decisions about HSCT. We are not sure, regardless of treatment, if this patient could have survived, but we hope that these patients can be captured for future research on hyperferritinemic sepsis.

## Author Contributions

Dr. EH cared for the patient in the case report and wrote the first draft. Drs. SR, JF, and SW assisted with the framing and additions of important references.

## Conflict of Interest Statement

The authors declare that the research was conducted in the absence of any commercial or financial relationships that could be construed as a potential conflict of interest.

## References

[1] Yamada K Yamamoto Y Uchiyama A Ito R Aoki Y Uchida Y Successful treatment of neonatal herpes simplex-type 1 infection complicated by hemophagocytic lymphohistiocytosis and acute liver failure. Tohoku J Exp Med (2008) 214(1):1–5. 10.1620/tjem.214.1 18212481

[2] Abuhasna SD Shihab ZM Al Niyadi SM Tatari HM Al Jundi AH Atwa KH. Neonatal herpes simplex fulminant hepatitis successfully treated with acyclovir. J Clin Neonatol (2012) 1(2):87–90. 10.4103/2249-4847.96761 24027697 PMC3743148

[3] Kotzbauer D Frank G Dong W Shore S. Clinical and laboratory characteristics of disseminated herpes simplex virus infection in neonates. Hosp Pediatr (2014) 4(3):167–71. 10.1542/hpeds.2013-0086 24785561

[4] Imashuku S Ueda I Teramura T Mori K Morimoto A Sako M Occurrence of haemophagocytic lymphohistiocytosis at less than 1 year of age: analysis of 96 patients. Eur J Pediatr (2005) 164(5):315–9. 10.1007/s00431-005-1636-9 15731905

[5] Henter JI Horne A Aricó M Egeler RM Filipovich AH Imashuku S HLH-2004: diagnostic and therapeutic guidelines for hemophagocytic lymphohistiocytosis. Pediatr Blood Cancer (2007) 48(2):124–31. 10.1002/pbc.21039 16937360

[6] Halstead ES Carcillo JA Schilling B Greiner RJ Whiteside TL. Reduced frequency of CD56 dim CD16 pos natural killer cells in pediatric systemic inflammatory response syndrome/sepsis patients. Pediatr Res (2013) 74(4):427–32. 10.1038/pr.2013.121 23857294

[7] Vladescu IA Browning WL Thomsen IP Massive ferritin elevation in neonatal herpes simplex virus infection: hemophagocytic lymphohistiocytosis or herpes simplex virus alone? J Pediatric Infect Dis Soc (2015) 4(3):e48–52. 10.1093/jpids/piv005 26407444

[8] Torti FM Torti SV Regulation of ferritin genes and protein. Blood (2002) 99(10):3505–16. 10.1182/blood.V99.10.3505 11986201

[9] Cohen LA Gutierrez L Weiss A Leichtmann-Bardoogo Y Zhang DL Crooks DR Serum ferritin is derived primarily from macrophages through a nonclassical secretory pathway. Blood (2010) 116(9):1574–84. 10.1182/blood-2009-11-253815 20472835

[10] Bennett TD Hayward KN Farris RW Ringold S Wallace CA Brogan TV. Very high serum ferritin levels are associated with increased mortality and critical care in pediatric patients. Pediatr Crit Care Med (2011) 12(6):e233–6. 10.1097/PCC.0b013e31820abca8 21263363

[11] Rosario C Zandman-Goddard G Meyron-Holtz EG D’Cruz DP Shoenfeld Y The hyperferritinemic syndrome: macrophage activation syndrome, still’s disease, septic shock and catastrophic antiphospholipid syndrome. BMC Med (2013) 11:185 10.1186/1741-7015-11-185 23968282 PMC3751883

[12] Risma K Jordan MB. Hemophagocytic lymphohistiocytosis: updates and evolving concepts. Curr Opin Pediatr (2012) 24(1):9–15. 10.1097/MOP.0b013e32834ec9c1 22189397

[13] Johnson TS Terrell CE Millen SH Katz JD Hildeman DA Jordan MB. Etoposide selectively ablates activated T cells to control the immunoregulatory disorder hemophagocytic lymphohistiocytosis. J Immunol (2014) 192(1):84–91. 10.4049/jimmunol.1302282 24259502 PMC4177106

[14] Demirkol D Yildizdas D Bayrakci B Karapinar B Kendirli T Koroglu TF Hyperferritinemia in the critically ill child with secondary hemophagocytic lymphohistiocytosis/sepsis/multiple organ dysfunction syndrome/macrophage activation syndrome: what is the treatment? Crit Care (2012) 16(2):R52. 10.1186/cc11256 22715953 PMC3681377

[15] Schulert GS Grom AA. Pathogenesis of macrophage activation syndrome and potential for cytokine-directed therapies. Annu Rev Med (2015) 66:145–59. 10.1146/annurev-med-061813-012806 25386930 PMC5846123

[16] Rajasekaran S Kruse K Kovey K Davis AT Hassan NE Ndika AN Therapeutic role of anakinra, an interleukin-1 receptor antagonist, in the management of secondary hemophagocytic lymphohistiocytosis/sepsis/multiple organ dysfunction/macrophage activating syndrome in critically ill children*. Pediatr Crit Care Med (2014) 15(5):401–8. 10.1097/PCC.0000000000000078 24583503 PMC7122718

[17] Sevketoglu E Yildizdas D Horoz OO Kihtir HS Kendirli T Bayraktar S Use of therapeutic plasma exchange in children with thrombocytopenia-associated multiple organ failure in the Turkish thrombocytopenia-associated multiple organ failure network. Pediatr Crit Care Med (2014) 15(8):e354–9. 10.1097/PCC.0000000000000227 25068251 PMC5287151

